# Beam Position Projection Algorithms in Proton Pencil Beam Scanning

**DOI:** 10.3390/cancers16112098

**Published:** 2024-05-31

**Authors:** Konrad P. Nesteruk, Stephen G. Bradley, Hanne M. Kooy, Benjamin M. Clasie

**Affiliations:** 1 Department of Radiation Oncology, Massachusetts General Hospital, Boston, MA 02114, USA; knesteruk@mgh.harvard.edu (K.P.N.); sgbradley@mgb.org (S.G.B.); hkooy@mgh.harvard.edu (H.M.K.); 2 Harvard Medical School, Boston, MA 02115, USA

**Keywords:** proton therapy, pencil beam scanning, beam position

## Abstract

**Simple Summary:**

Pencil beam scanning nozzles monitor the beam position in real time and record the results in log files. We cannot, however, place a beam position monitor at the isocenter during treatment, so accurate online beam position corrections and log file analyses rely on an algorithm to project the beam position from the nozzle to the isocentric plane. We present four generic algorithms and determined the accuracy of each approach in three example configurations and two nozzle lengths.

**Abstract:**

Beam position uncertainties along the beam trajectory arise from the accelerator, beamline, and scanning magnets (SMs). They can be monitored in real time, e.g., through strip ionization chambers (ICs), and treatments can be paused if needed. Delivery is more reliable and accurate if the beam position is projected from monitored nozzle parameters to the isocenter, allowing for accurate online corrections to be performed. Beam position projection algorithms are also used in post-delivery log file analyses. In this paper, we investigate the four potential algorithms that can be applied to all pencil beam scanning (PBS) nozzles. For some combinations of nozzle configurations and algorithms, however, the projection uses beam properties determined offline (e.g., through beam tuning or technical commissioning). The best algorithm minimizes either the total uncertainty (i.e., offline and online) or the total offline uncertainty in the projection. Four beam position algorithms are analyzed (A1–A4). Two nozzle lengths are used as examples: a large nozzle (1.5 m length) and a small nozzle (0.4 m length). Three nozzle configurations are considered: IC after SM, IC before SM, and ICs on both sides. Default uncertainties are selected for ion chamber measurements, nozzle entrance beam position and angle, and scanning magnet angle. The results for other uncertainties can be determined by scaling these results or repeating the error propagation. We show the propagation of errors from two locations and the SM angle to the isocenter for all the algorithms. The best choice of algorithm depends on the nozzle length and is A1 and A3 for the large and small nozzles, respectively. If the total offline uncertainty is to be minimized (a better choice if the offline uncertainty is not stable), the best choice of algorithm changes to A1 for the small nozzle for some hardware configurations. Reducing the nozzle length can help to reduce the gantry size and make proton therapy more accessible. This work is important for designing smaller nozzles and, consequently, smaller gantries. This work is also important for log file analyses.

## 1. Introduction

Proton therapy is a radiation therapy modality that allows treating patients with a millimeter accuracy. This is made possible by the unique properties of protons, which deposit most of their energy at the end of their penetration range [1]. The maximum occurs at the Bragg peak, after which the energy deposition rapidly decays to zero. This allows for the effective sparing of surrounding healthy tissue and organs at risk and thus reducing treatment toxicity. Proton therapy is routinely used for the treatment of various tumor sites [2,3,4,5,6,7,8,9,10,11,12,13,14,15,16,17,18,19] and is a dynamically developing form of radiotherapy [20].

The current state-of-the-art dose delivery technique in proton therapy is pencil beam scanning (PBS) [21,22,23]. In PBS, narrow pencil beams are scanned across the tumor in three dimensions. It achieves highly conformal dose distributions, maximizing the dose to the target while minimizing the exposure to the surrounding healthy tissue. This technique requires a very high level of precision in the position of each pencil beam.

Beam position is measured by strip ionization chambers (ICs) and other instruments in the PBS nozzle [24] and beamline. Beam position uncertainties can arise from the accelerator, beamline, and scanning magnets (SMs). While clinical physicists are concerned about the beam position at the isocenter, ICs cannot be used for direct monitoring at this location during treatments: a projection is needed.

Beam position uncertainty should not exceed ±0.4 mm (at the isocenter) for a beam of width (one sigma) 3 mm to achieve a dose uniformity of ±3% [25]. This is hard to achieve in practice. While the analysis in TG-224 is for a single, uniform layer, there is a similar implication when there is the relative lateral movement of non-uniform-weight spots between layers. Online beam position corrections (i.e., during treatment) use information from the ICs and other feedback to correct the trajectory. A small amount of beam is delivered to the patient to allow the beam position to be measured. The beam current may or may not be reduced to zero depending on the required correction. An accurate projection algorithm determines the position correction at the isocenter, which is converted into an adjustment of the scanning magnets (SMs) or other beam steering mechanism.

Log file analyses examine the proton therapy machine parameters after the delivery of a beam. These contain the IC and SM feedback that may be used to project the beam position to the isocenter (the projection may be performed by the manufacturer, but, in any case, a projection is required to obtain beam properties in the isocentric plane). Log files may be used as part of a quality assurance (QA) program and to monitor treatment accuracy. Log file analyses benefit from having information from all spots in the beam at once, so the analysis can more easily combine nozzle feedback from multiple spots for more information. An accurate projection algorithm is essential for these analyses.

There are similar publications related to this work. Giordanengo and Donetti 2018 [24] described pencil beam scanning nozzles and instrumentation. They recommend performing online position corrections. Psoroulas et al. 2018 [26] discussed beam position corrections; they characterized the effect of the settling of the beamline after energy changes and applied this calibrated correction to the beginning of each layer. Li et al. 2013 [27] performed a log file analysis using a position projection algorithm that assumes the unscanned beam is well aligned with the isocenter. Their projection assumes all uncertainties come from the SMs. They justified this approach, as the alignment of an unscanned beam with the isocenter was checked offline during QA, with the results being better than ±0.1 mm. Lin et al. 2017 [28] used the online beam position measured both upstream and downstream of the SM in the projection, as well as performed the online calibration of the SM deflection angle determined from multiple spot positions on the downstream IC vs. the SM current. Tan et al. 2023 [29] used a position projection algorithm similar to the one described by Li et al. 2013 [27].

While particle therapy vendors have used online beam position corrections to improve delivery accuracy, these algorithms are proprietary. Most are thought to be similar to the algorithm in Li et al. 2013 [27].

At present, it is unclear if the algorithms described above are equivalent, if there are more types of projections, and which algorithm is best. In this paper, we describe four projection algorithms that can be applied to all PBS nozzles and explain why they are different. For some combinations of nozzle configuration and algorithm, however, the projection algorithm must use the beam properties determined offline (e.g., through beam tuning, QA, or technical commissioning). We call these offline uncertainties. We determined the performance of each algorithm with example configurations and uncertainties and defined the best algorithm as the one that minimized either the total uncertainty (i.e., online and offline) or the total offline uncertainty in the projection. This work is important for accurate beam position corrections and log file analyses.

Throughout this paper, nozzle configuration describes the configuration of the ICs, which measure beam position, and SMs, which deflect the beam trajectory. SMs can be two separate magnets or a single combined-function magnet that scans in two axes. ICs may be before, after, or between SMs, or some combination thereof. Algorithm describes the method and assumptions used to project the position to the isocenter.

## 2. Materials and Methods

### 2.1. Beam Trajectory in a PBS Nozzle

**Figure 1:**
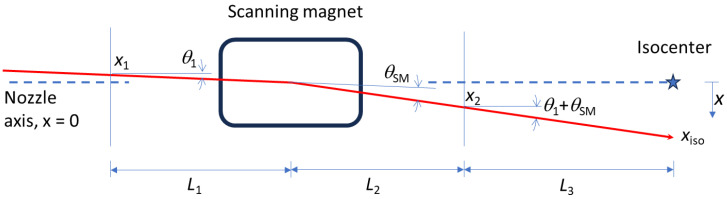
Trajectory of a proton beam (red, solid line) through a scanning nozzle. A single scanning axis, the *x* axis, is shown for simplicity. Isocenter is the blue star. The nozzle axis (blue dashed line) represents the line with *x* = 0. The beam trajectory is an arc within the scanning magnet, but it is shown as a single bend at the center of the scanning magnet. The vertical blue lines represent planes in space that may or may not correspond to positions of ICs.

Figure 1 shows a proton beam trajectory (in red) represented by 8 variables (*x*_1_, *x*_2_, *x*_iso_, *θ*_1_, *θ*_SM_, *L*_1_, *L*_2_, and *L*_3_). Our goal was to obtain *x*_iso_ by projection. Three variables, the lengths *L*_1_, *L*_2_, and *L*_3_, can be measured through mechanical means and/or technical commissioning and do not change. A further 2 of the 8 variables are dependent variables through the trigonometric relationships

(1) $$x_{2}=x_{1}+L_{1}\tan\theta_{1}+L_{2}\tan\left(\theta_{1}+\theta_{SM}\right),$$

and

(2) $$x_{iso}=x_{2}+L_{3}\tan\left(\theta_{1}+\theta_{SM}\right).$$

Obviously, *x*_iso_ is one of these dependent variables. One remaining variable can be eliminated from the projection algorithm as it is dependent on the other variables according to Equation (1). We define this as the eliminated variable. Eliminating a variable through Equation (1) reduces the uncertainties in the projection, but care must be taken in the selection.

There are three remaining variables that need to be constrained. These may be constrained by online beam position measurements at strip ionization chambers, inferred from online scanning magnet setpoint or feedback, beam pipe diameter, prior retractable beam position monitoring results (if they are repeatable), collimator location (if present), or repeatable beam properties from technical commissioning. We define these as the constrained variables.

We treat *θ*_SM_ as an online variable, which is obtained from a calibration against the SM setpoint or Hall probe feedback of the magnetic field. The calibration assumes, as is usually the case, that the effects from the iron core saturation, X–Y scanning magnet coupling, and hysteresis are small or can be corrected. This calibration can be obtained directly and verified in a postdelivery analysis, where the change in *θ*_SM_ is known for all spots against the change in setpoint or Hall probe feedback in a constant energy layer.

### 2.2. Algorithm A1: x_1_, x_2_, and θ_SM_ Are Constrained Variables; θ_1_ Is the Eliminated Variable

We can solve for *θ*_1_ by expanding tan(*θ*_1_ + *θ*_SM_) = (tan*θ*_1_ + tan*θ*_SM_)/(1 − tan*θ*_1_tan*θ*_SM_) in Equation (1), which gives, after some manipulation

$$a\;\tan^{2}\theta_{1}+b\tan\theta_{1}+c=0,$$

where

$$\begin{array}{ll} a & =-L_{1}\tan\theta_{SM}\\ b & =\left(x_{2}-x_{1}\right)\tan\theta_{SM}+L_{1}+L_{2}\\ c & =-\left(x_{2}-x_{1}\right)+L_{2}\tan\theta_{SM} \end{array}$$

This can have two solutions for tan(*θ*_1_) from the quadratic formula (note the angle of the beam in the *L*_2_ region is *θ*_1_ + *θ*_SM_, not *θ*_SM_ alone). Assume *θ*_1_ and *θ*_SM_ are small, *L*_1_ and *L*_2_ are of the order of 100 to 1000 mm, and *x*_2_ and *x*_1_ are of the order of 1 to 10 mm, so *b* >> *a* and *b* >> *c.* The positive solution of the quadratic formula is simplified using a Taylor expansion, giving

$$\begin{array}{ll} \tan\theta_{1} & =\left(-b+\sqrt{b^{2}-4ac}\right)/2a\\ & =(-b+b(1-2ac/b^{2}+\ldots ))/2a\approx -c/b \end{array}$$

The negative quadratic solution is ignored as the magnitude of *b* would overwhelm the numerator in the Taylor series expansion, resulting in a large tan(*θ*_1_). Using the small angle approximation, tan(*θ*_1_) ≈ *θ*_1_ and tan(*θ*_SM_) ≈ *θ*_SM_, we obtain

$$\theta_{1}\approx \frac{\left(x_{2}-x_{1}\right)-L_{2}\theta_{SM}}{{\left(x_{2}-x_{1}\right)\theta }_{SM}+L_{1}+L_{2}}\approx \frac{\left(x_{2}-L_{2}\theta_{SM}\right)-x_{1}}{L_{1}+L_{2}}.$$

Intuitively, *x*_2_ − *L*_2_*θ*_SM_ is the approximate location of the trajectory at the second position if the scanning magnet deflection is subtracted. This equation represents the slope of the remaining straight line from position 1 to position 2. The beam position at the isocenter is then obtained from Equation (2)

(3) $$x_{iso}\approx x_{2}+L_{3}\theta_{SM}+\frac{L_{3}\left(x_{2}-L_{2}\theta_{SM}-x_{1}\right)}{L_{1}+L_{2}}.$$

### 2.3. Algorithm A2: x_1_, x_2_, and θ_1_ Are Constrained Variable, θ_SM_ Is the Eliminated Variable

We can solve for *θ*_SM_ by expanding tan(*θ*_1_ + *θ*_SM_) in Equation (1), giving

$$\tan\left(\theta_{SM}\right)=\frac{x_{2}-x_{1}-\left(L_{1}+L_{2}\right)\tan\left(\theta_{1}\right)}{\left(x_{2}-x_{1}-L_{1}\tan\theta_{1}\right)\tan\left(\theta_{1}\right)+L_{2}}.$$

Assuming *θ*_1_ and *θ*_SM_ are small, the result is

$$\theta_{SM}\approx \frac{x_{2}-\left(L_{1}+L_{2}\right)\theta_{1}-x_{1}}{L_{2}}.$$

Intuitively, *x*_2_ − (*L*_1_ + *L*_2_)*θ*_1_ is the approximate location of the trajectory at the second position if the beam angle upstream of the scanning magnet is zero. This equation represents the slope of the remaining straight line from the center of the scanning magnet to position 2. The beam position at the isocenter is then obtained from Equation (2)

(4) $$x_{iso}\approx x_{2}+L_{3}\theta_{1}+\frac{L_{3}\left(x_{2}-\left(L_{1}+L_{2}\right)\theta_{1}-x_{1}\right)}{L_{2}}.$$

### 2.4. Algorithm A3: x_2_, θ_SM_, and θ_1_ Are Constrained Variables; x_1_ Is the Eliminated Variable

This is a trivial algorithm that represents a straight-line projection. Equation (2) can be solved with the constrained variables. Assuming *θ*_1_ and *θ*_SM_ are small, the result is

(5) $$x_{iso}\approx x_{2}+L_{3}{(\theta }_{1}+\theta_{SM}).$$

### 2.5. Algorithm A4: x_1_, θ_SM_, and θ_1_ Are Constrained Variables; x_2_ Is the Eliminated Variable

This represents a design that is generally not used clinically in PBS nozzles, as there is almost always a strip ionization chamber at position 2. For completeness, however, the solution for this scenario is given by substituting Equation (1) into Equation (2), giving

$$x_{iso}=x_{1}+L_{1}\tan\theta_{1}+L_{2}\tan\left(\theta_{1}+\theta_{SM}\right)+L_{3}\tan\left(\theta_{1}+\theta_{SM}\right).$$

Assuming *θ*_1_ and *θ*_SM_ are small, the result is

(6) $$x_{iso}\approx x_{1}+L_{1}\theta_{1}{+L}_{2}\left(\theta_{1}+\theta_{SM}\right)+L_{3}\left(\theta_{1}+\theta_{SM}\right)$$

### 2.6. Nozzle Configuration

The possible IC configurations are as follows:

- After = IC downstream of SM;
- Before = IC upstream of SM.

PBS nozzles may contain one or both configurations. Each axis is considered separately, so each axis may have a different configuration.

### 2.7. Error Analysis

The default uncertainties and lengths are given in Table 1 and Table 2. Uncertainties are propagated to *x*_iso_ using standard techniques for uncorrelated uncertainties (see, for example, Ref. [30]). For variable *F*, which is a linear combination of uncorrelated variables *A* and *B*, with standard deviations *σ_A_* and *σ_B_*_,_ it holds that

$$F=\alpha A+\beta B\;\;\Rightarrow \;\;\sigma_{F}^{2}=\alpha^{2}\sigma_{A}^{2}+\beta^{2}\sigma_{B}^{2},$$

where α and β are constants, and *σ_F_* is the standard deviation of variable *F*. All results use small angle algorithms (Equations (3)–(6)), which are linear in the constrained variables.

## 3. Results

Table 3 shows the contribution to the total uncertainty in *x*_iso_ from each of the uncertainties for each algorithm. Table 4 shows the total offline uncertainties for each nozzle configuration and algorithm.

**Figure 2:**
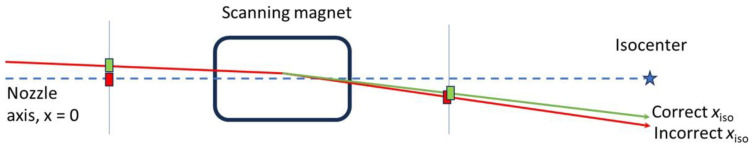
An example of the nozzle position interlocks changing due to an SM correction, with ICs before and after the SM. The blue dashed line is the nozzle axis and the blue star is isocenter. The red arrow shows the initial trajectory of the beam and nozzle position interlock windows on the ICs as red bands. After an SM correction, the beam position at the isocenter is moved to the green arrow. The IC interlocks may not be satisfied after this beam position correction, so the interlock windows may need to change to be better centered on the corrected trajectory (green bands).

Some algorithms may need the nozzle position interlock windows to change after a position correction (Figure 2). The nozzle could incorporate trim magnets installed upstream in the beamline and steer the beam online on IC before the SM. The advantages of this technique are (1) the nozzle position interlocks do not need to move after such a correction, and (2) the *θ*_1_ uncertainty may be smaller (depending on the phase advance from the trim magnets to the upstream IC).

**Figure 3:**
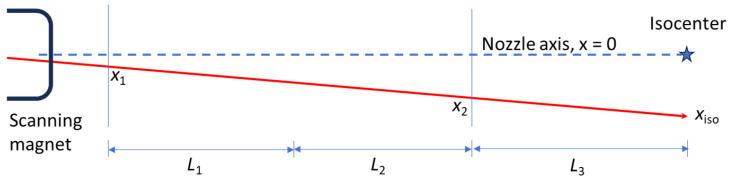
An example of a nozzle configuration where an IC is between scanning magnets. The axis where the scanning magnet is upstream of both ICs is shown. This axis has a straight-line projection from position 1 to position 2 to the isocenter.

A nozzle configuration with an IC between scanning magnets would result in one axis having no scanning magnet between ICs. This is shown in Figure 3. This is the trivial case and Equation (2) can be applied directly (see Algorithm A3), giving

(7) $$x_{iso}=x_{2}+L_{3}\left(x_{2}-x_{1}\right)/(L_{1}+L_{2}),$$

and there are no offline variables. The propagation of errors is given in Table 5.

## 4. Discussion

**Figure 4:**
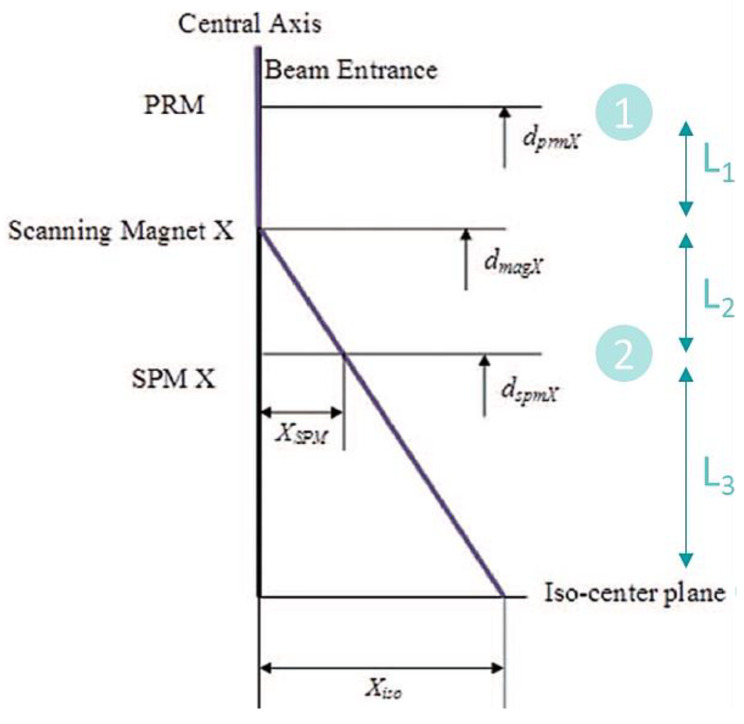
The projection algorithm used in Refs. [27,29], with $x_{iso}=x_{2}\times (L_{3}+L_{2})/L_{2}$. The planes marked as “1” and “2” correspond to the labels used in Figure 1. If the scanning magnet deflection angle is incorrect, the projection is still accurate. This is, therefore, an example of Algorithm A2. The offline variables in this example are *x*_1_ and *θ*_1_, which are set to zero. If these variables are not zero during a delivery, the projection algorithm would be inaccurate. The figure is adapted from Ref. [27].

Refs. [27,29] used Algorithm A2 in their log file analyses (see Figure 4). Intuitively, one can see this is the case if *θ*_SM_ is incorrect. This does not affect the accuracy of the projection, so *θ*_SM_ is the eliminated variable. This can be a good choice for a projection algorithm when the uncertainty in *θ*_SM_ is large. Ref. [28] used Algorithm A1 in their log file analyses. Similarly, one can see this is the case by considering the case if *θ*_1_ is incorrect. This does not affect the accuracy of the projection, so *θ*_1_ is the eliminated variable. Algorithm A1 is usually a good choice for large nozzles as the total uncertainty and total offline uncertainty are small in Table 3 and Table 4.

The small nozzle configuration in Table 2, with a nozzle length of only 400 mm, may not allow enough space for two separate scanning magnets. This situation may arise in a single axis if there is an ion chamber between the SM (the other axis will look like that in Figure 3). It may also arise if the nozzle has a combined-function SM.

The limitations of this study include the limited set of uncertainties listed in Table 1 and the dimensions in Table 2. The results for other values can be obtained by scaling as appropriate or reanalyzing using the methods described above. Also, uncertainties in the lengths L_1_, L_2_, and L_3_ were not considered, and these may be important for extremely compact nozzles. Uncertainties in these parameters can be propagated to *x*_iso_ using the methods described above.

Other considerations for designing a nozzle and selecting an algorithm may include the following:

- The small nozzle has larger total uncertainty than the large nozzle for the best algorithm in each scenario, largely due to the some of the demagnifications from the longer lengths between constrained positions. Instrumentation in smaller nozzles may need to be more precise to achieve the same level of uncertainty in the projection.
- ICs far from the isocenter contribute more to multiple-Coulomb scattering (MCS) which increases the pencil beam size.
- Online position correction algorithms should remember the prior correction after each spill and/or layer change until the end of the beam, and the beam current should be modulated to zero while the SM correction is being applied and turned back on automatically. This can be seen to be optimal by considering random and systematic position uncertainties. In the case of random uncertainties, there are pauses with each spill and/or layer change independent of the approach used. In the case of systematic position uncertainties, there is only one position correction needed if the prior correction is remembered and applied for the whole beam.
- Tuning pulses may help to make a more accurate position projection. These tuning pulses can be delivered with the scanning magnets set to zero, with the intention of centering the beam without *θ*_SM_ in the projection, and then adding scanning afterward. The projection in this case would be similar to that shown in Figure 3. While this method can help reduce uncertainties, care should be taken to make sure the effect of residual magnetism in the scanning magnets is negligible, and the amount of extra dose delivered to the patient is negligible.
- There must be redundant devices or checks on the performance of the nozzle instruments when performing online beam position corrections.

## 5. Conclusions

Often the best choice of algorithm is simply the one that eliminates the variable with the highest uncertainty, usually by focusing on the variable measured by the least reliable device or not measured. That may not always be the best choice if a variable is demagnified in the projection. This also explains why the best algorithm can change depending on the nozzle size, as the uncertainties can be magnified in different ways.

We defined the best algorithm to use as the one with the smallest total uncertainty or the smallest total offline uncertainty, depending on what needs to be minimized. For large and small nozzles, the smallest total uncertainty is obtained with Algorithms A1 and A3, respectively. The total offline uncertainty can be important; if it is large, it may put extra burden on QA and machine recommissioning. For the large nozzle, the smallest total offline uncertainty is obtained with Algorithm A1. For the small nozzle, the smallest total offline uncertainty changes between Algorithms A1 and A3, depending on the nozzle configuration, and if *θ*_SM_ is an offline variable.

Reducing the nozzle length can help to reduce the gantry size and make proton therapy more accessible. Prior experience with beam position projection algorithms for large nozzles may no longer be optimal for small nozzles. This finding is important for designing smaller nozzles and, consequently, smaller gantries. This work is also important for log file analyses.

## Figures and Tables

**Table 1:** Default uncertainties in an example trajectory.

Parameter	Uncertainty (1 σ)
*x*_1_	0.5 mm
*x*_2_	0.5 mm
*θ*_1_	1 mrad
*θ*_SM_	1 mrad

**Table 2:** Default lengths in large and small proton PBS nozzles.

Parameter	Large Nozzle (mm)	Small Nozzle (mm)
*L*_1_	500	200
*L*_2_	1000	200
*L*_3_	500	500

**Table 3:** Contributions to the total uncertainty in *x*_iso_ from each of the uncertainties. Algorithms A1–A4 are defined in Section 2. Some uncertainties do not affect *x*_iso_; e.g., *θ*_1_ is eliminated from the projection in Algorithm A1 (see Equation (3)), so it has zero contribution. For the large and small nozzles, the smallest total uncertainty is from Algorithms A1 and A3, respectively. The most appropriate algorithm for each configuration is shown in bold.

Uncertainty	Uncertainties (1 σ) Projected to *x*_iso_ [mm] for Algorithms A1–A4							
	Large Nozzle				Small Nozzle			
	A1	A2	A3	A4	A1	A2	A3	A4
*θ*_1_	0	0.25	0.5	2	0	0.5	0.5	0.9
*θ*_SM_	0.17	0	0.5	1.5	0.25	0	0.5	0.7
*x*_1_	0.17	0.25	0	0.5	0.63	1.25	0	0.5
*x*_2_	0.67	0.75	0.5	0	1.13	1.75	0.5	0
Total	**0.71**	0.83	0.87	2.55	1.31	2.21	**0.87**	1.24

**Table 4:** Total offline uncertainties for each nozzle configuration and algorithm. The total offline uncertainty when *θ*_SM_ is included as an offline variable (which may be appropriate for some nozzles) is given in round brackets. For the large nozzle, the smallest total offline uncertainty is from Algorithm A1. For the small nozzle, the smallest total offline uncertainty changes between Algorithms A1 and A3 depending on the nozzle configuration and if *θ*_SM_ is an offline variable. The most appropriate algorithm for each configuration is shown in bold.

Nozzle Configuration	Offline Variables	Total Offline Uncertainty (1 σ) in *x*_iso_ [mm] for Algorithms A1–A4							
		Large Nozzle				Small Nozzle			
		A1	A2	A3	A4	A1	A2	A3	A4
IC Before and After	*θ*_1_ (+*θ*_SM_)	**0** **(0.17)**	0.25 (0.25)	0.5 (0.71)	2 (2.50)	**0** **(0.25)**	0.5 (0.50)	0.5 (0.71)	0.9 (1.14)
IC After	*θ*_1_, *x*_1_ (+*θ*_SM_)	**0.17** **(0.24)**	0.35 (0.35)	0.5 (0.71)	2.06 (2.55)	0.63 **(0.67)**	1.35 (1.35)	**0.5** (0.71)	1.03 (1.24)
IC Before	*θ*_1_, *x*_2_ (+*θ*_SM_)	**0.67** **(0.69)**	0.79 (0.79)	0.71 (0.87)	2 (2.50)	1.13 (1.15)	1.82 (1.82)	**0.71** **(0.87)**	0.9 (1.14)

**Table 5:** Total offline and total uncertainties for the projection in Figure 3.

Uncertainty	Large Nozzle	Small Nozzle
*x*_1_	0.17	0.63
*x*_2_	0.67	1.13
Total offline	0	0
Total	0.69	1.29

## Data Availability

The data presented in this study are available in this article.
